# “They pulled that funding away and we’re not recovering. it’s getting worse”: deaths of despair in post-austerity north east England

**DOI:** 10.1186/s12939-024-02334-w

**Published:** 2024-11-19

**Authors:** Timothy Price

**Affiliations:** Population Health Sciences Institute, Faculty of Medical Sciences, Newcastle University, NE1 4LP, Newcastle upon Tyne, UK

## Abstract

**Background:**

Deaths related to suicide, drug misuse, and alcohol-specific causes, known collectively as “deaths of despair” are of growing interest to researchers in England. Rates of death from these causes are highest in deprived northern communities and are closely tied to the social determinants of health and the policy decisions that have shaped them. The aim of this paper is to explore how stakeholders and community members living in Middlesbrough and South Tyneside, two Northern towns with above average rates of deaths of despair, understood the relationship between austerity policies and rates of deaths from these causes in their areas.

**Methods:**

I conducted interviews and one focus group with a total of 54 stakeholders and community members in Middlesbrough and South Tyneside. Data were analysed using the iterative categorisation technique and the findings were interpreted through thematic analysis.

**Results:**

The findings highlight four primary ways through which austerity exacerbated rates of deaths of despair in Middlesbrough and South Tyneside: reduced access to mental health services, diminished substance abuse treatment capacity, loss of youth services, and the closure of community institutions. Participants linked these cuts to rising social isolation, declining mental health, and increased substance misuse, which collectively deepened geographic inequalities in deaths of despair.

**Conclusions:**

This study underscores the urgent need for reinvestment in local services to reduce inequalities and prevent further unnecessary deaths due to drug, suicide, and alcohol-specific causes. Prioritising the restoration and enhancement of services lost to austerity is critical. Such reinvestment will not only help to alleviate some of the most immediate need but also form a foundation for addressing the wider structural inequalities that perpetuate deaths of despair.

## Background

‘Deaths of despair’ (DoD), those due to drug, suicide, and alcohol-specific mortality, have been the subject of growing academic interest since 2015, when Case & Deaton observed that deaths from these causes among middle-aged non-Hispanic Whites were driving a decline in US life expectancy [1, 2]. They later proposed that cumulative economic and social disadvantage caused by worsening labour market and social conditions had given rise to a sense of despair and ultimately increased the likelihood of DoD [3]. While drug, suicide, and alcohol-specific deaths have different underlying causes, the relationship between them is complex and overlapping. Alcohol and drug abuse are known risk factors for suicide [4, 5] and drug overdoses often involve the use of both drugs and alcohol prior to death, a phenomenon known as polysubstance abuse [6, 7], demonstrating how closely intertwined morbidity and mortality from these causes are and justifying examining them as a unified phenomenon.

Much of the research surrounding DoD has been conducted in the United States (US), but there is growing interest in deaths from these causes in other high-income countries. In the United Kingdom (UK), life expectancy stagnated prior to the COVID-19 pandemic, in part due to increased DoD [8]. DoD in the UK have risen since the mid-2000s and have primarily affected people of middle age [9]. Case and Deaton proposed that cumulative economic disadvantage progressively increased the risk of DoD throughout the life course, leading to the prevalence of these deaths in middle-aged populations [2, 3]; subsequent research has affirmed their proposition [10–12]. Increases in DoD have primarily been driven by significant increases in drug-related mortality, with alcohol-specific mortality and deaths by suicide increasing at a lower rate [10, 13]. Within the UK, rates of DoD follow clear geographic patterns; for example, people in Scotland bear a higher burden of DoD than their counterparts in other constituent countries [9, 14]. Within England, northern regions experience a significantly higher burden of DoD than other regions and inequalities between different towns and cities persist within regions [13, 15]. In the North East, the English region with the highest rate of DoD mortality, Middlesbrough and South Tyneside hold significantly higher than average rates of drug, suicide, and alcohol-related mortality, although the rates of these deaths vary between these two towns [16–18].

The literature surrounding the factors driving the rise in DoD continues to develop, with structural factors such as labour market, housing, and welfare policy associated with rates of DoD [19–22]. There are also associations between increased rates of DoD with deprivation and economic decline, as deaths from these causes often concentrate in deindustrialised, low-income areas such as the US Rust Belt [23, 24], former industrial areas of Eastern Europe [12, 25], and North East England [13, 15]. It is clear from the available evidence that rates of DoD are closely tied to the social determinants of health, the conditions in which people are born, live, work, and age [26]. To understand the forces driving rates of DoD in the UK therefore, one must look to the factors that have influenced the social determinants of health in that context.

One such factor that has shaped the social determinants of health in England in recent years are the post-2008 austerity policies [27]. Introduced in response to a growing national budget deficit as a result of the 2008 Global Financial Crisis, austerity measures reduced local authority budgets by 30% between 2008 and 2015 and led to the shuttering of many public services [28]. Simultaneously, welfare reform measures most severely impacted the poorest areas in the country (where a higher proportion of the population received support), many of which were communities in the North East like Middlesbrough and South Tyneside [27, 29]. The worst-hit local authority areas – largely located in the North – lost around four times as much, per adult of working age, as the authorities least affected by austerity – found entirely in the South and East of England (e.g. Hart, Hampshire) [29]. In this study, participants provided insight into how austerity policies had shaped day-to-day life in their areas and helped create the social environment in which DoD occur.

Participants in this study were recruited from two local authorities in North East England, Middlesbrough and South Tyneside. These recruitment sites were selected based on the above-average rates of DoD in these communities. Table 1 shows age-standardised mortality rates for DoD in Middlesbrough and South Tyneside in comparison with regional and national averages.

**Table 1:** Three-year average (2020-22) age-standardised mortality rates from drug, suicide, and alcohol-specific mortality per 100,000 population. All data from ONS [16, 17, 30]

	Middlesbrough	South Tyneside	North East	England
Suicide	16.5	8.9	13.5	10.3
Drug Misuse Deaths	14.1	10.9	9.7	5.2
Alcohol-Specific Mortality	26.3	28.3	21.8	14.5

## Methods

### Recruitment

Recruitment was carried out in two phases; phase one targeted stakeholders, while phase two targeted community members. Stakeholders were eligible to participate in this study if their work involved people living in Middlesbrough or South Tyneside and if their work pertained directly or indirectly to drug, self-harm, or alcohol-specific morbidities and mortalities. Several purposive sampling techniques were used to recruit stakeholders. A short description of the study and an invitation to participate was distributed through a mailing list for stakeholders in Middlesbrough. Individual stakeholders were also approached directly via their publicly available email addresses and invited to participate if they worked in a sector that had been frequently discussed in previous interviews but was as yet unrepresented in the sample. Snowball sampling of stakeholders’ networks was used to further the reach of recruitment materials. The sample of stakeholders consisted of 24 people. Table 2 presents stakeholder demographic information.

**Table 2:** Stakeholder demographic information

Category	Middlesbrough (Number of Participants)	South Tyneside (Number of Participants)
Gender		
Male	6	7
Female	7	4
Age		
18–24	0	1
25–34	1	1
35–44	1	3
45–54	7	4
55–64	4	2
Highest Level of Education		
Higher or secondary or further education (A-levels, BTEC, etc.)	4	2
University	6	6
Postgraduate Degree	2	3
Prefer not to say	1	0
Years in Current Professional Role		
Less than 1 Year	4	2
1 to 2 Years	3	0
2 to 5 Years	0	4
5 to 10 Years	1	4
More than 10 Years	4	1
Prefer not to say	1	0
Total Participants	13	11

Community members were eligible to participate if they were over the age of 18, able to complete an interview in English, were a resident of Middlesbrough or South Tyneside, and were comfortable discussing DoD. The goal of this study was to learn how people who lived and worked in Middlesbrough and South Tyneside understood the determinants of the above-average rates of DoD in their towns, and not the personal factors influencing individual causes of despair and death. To that end, while anyone eligible was welcome to participate, participants were not required to have been directly impacted by DoD (e.g. through the loss of a close friend or family member) to be eligible to participate and no specific efforts were made to recruit from that population. In both towns, I advertised the study by displaying flyers in community spaces (e.g. libraries and community centres) and through engagement with key stakeholders that had access to the public (i.e. food banks, welfare-to-work organisations, and housing providers). I identified several community drop-ins in Middlesbrough and South Tyneside that were open to the public. These groups were aimed at creating opportunities for socialisation and peer connection in the interest of promoting general health and wellbeing, rather than providing support for specific issues (as would be the case for formal peer-support groups like alcoholics Anonymous). The organisations responsible for hosting the drop-ins were contacted to request permission for me to attend and to recruit participants from their group. Once permission had been granted, I attended these groups regularly. After building rapport with the group, I asked any group members interested in participating to schedule an interview. All the attendees at the drop-ins were invited to participate. The sample of community members consisted of 30 people. Table 3 presents community member demographic information

**Table 3:** Community member demographic information

Category	Middlesbrough (Number of Participants)	South Tyneside (Number of Participants)
Gender		
Male	9	9
Female	7	5
Age		
18–24	1	0
25–34	2	1
35–44	2	0
45–54	3	1
55–64	3	8
65+	5	4
Highest Level of Education Completed		
Primary school	5	2
Secondary school up to 16 years	4	8
Higher or secondary or further education (A-levels, BTEC, etc.)	2	3
College, university, post-graduate degree	2	1
Prefer not to say	3	0
Employment Status		
Unemployed	7	9
Part-Time	2	0
Full-Time	2	1
Retired	5	4
Years Lived in Town		
1 to 5 Years	1	1
6 to 10 Years	2	3
11 to 15 years	2	0
15+	11	10
Total Participants	16	14

### Data collection and analysis

Data were collected through semi-structured, in-depth interviews guided by a bespoke topic guide. Community members from one community drop-in requested to be interviewed together, to accommodate that request, data was collected from these participants (*n* = 6) via a focus group. The topic guide (see supplementary material) for the interviews and the focus group was informed by a review of the existing literature around DoD morbidity and mortality and was loosely modelled after a discussion guide previously used in a qualitative study investigating communal perceptions of diseases of despair in the US [31]. Participants were asked about how a range of factors, from individual mental health to socioeconomic deprivation, and government policy may contribute to deaths of despair. This paper explores participants’ perceptions of how austerity policies worsened geographic inequalities in DoD. Findings unrelated to the effects of austerity on geographic inequalities in DoD are reported elsewhere.

Interviews were conducted online using Microsoft Teams, in-person at the participants’ places of work, or in a public setting such as a coffee shop or community centre according to participants’ preferences. Stakeholders worked in a range of professional backgrounds including law enforcement, the voluntary and community sector, charity service provision (such as foodbanks and homeless outreach), mental health treatment, substance abuse recovery support, community organising, local government, public health, and housing management. Most community members had lived in their town for more than five years, were over the age of 45, and were either unemployed or retired. Gathering data from a stakeholders from a range of professional backgrounds and longtime residents elicits valuable nuance and multiple insights by incorporating a broad range of perspectives and expertise, which enhances the depth and comprehensiveness of the findings. All interviews and the focus group were audio recorded (with consent) and transcribed by the researcher, with identifying information (e.g. names and areas of residence) removed. Community members were provided with a £25 supermarket voucher to thank them for participating. Stakeholders were not provided with a voucher as their interviews were completed during their regular working hours. Data collection stopped when I deemed that data saturation had been reached i.e., that continuing to generate additional data would not yield additional relevant themes [32].

Data analysis was conducted using the Iterative Categorization (IC) technique developed by Neale [33] and findings were interpreted through thematic analysis [34]. IC is a technique for analysing qualitative data that was first published in 2016 and has previously been used to support qualitative research investigating addiction [35, 36]. IC is not a stand-alone method of analysing qualitative data; it is a technique for managing data analysis that is rigorous and transparent while remaining compatible with other common forms of qualitative analysis, such as thematic analysis. Coding was conducted using the qualitative analysis software MAXQDA 2022 [37]. An initial coding matrix was generated deductively based on the interview topic guide; codes were merged, and the matrix was supplemented with codes generated inductively as coding progressed. These codes were used to identify the specific policies and time periods that participants identified as driving DoD in their areas. The coding matrix and a sub-sample of coded transcripts were reviewed by another researcher within the author’s research institute to ensure reliability and rigour. Once coding was completed, the analysis followed the stages of IC outlined by Neale [33, 38] (.

The final stage of IC, interpretive analysis, seeks to identify patterns, associations, and explanations within the data [38]. Interpretive analysis involves identifying themes that appear in the data and exploring how these themes corroborate, expand, or refute existing constructs and theories. Interpretive analysis within IC involves three processes: conceptualising, differentiating, and externalising. In this study, conceptualising was undertaken inductively and involved the identification of five themes within the data relating to how austerity had worsened regional inequalities in DoD. Data differentiation involves checking descriptive themes and categories for similarities, differences, and outliers within participant accounts. Differentiation of participant accounts is conducted based on inclusion in subgroups and characteristics relevant to the study; in this case, participants were differentiated based on gender, area of residence, and their status as a stakeholder or community member. Themes were differentiated to investigate whether participants who expressed similar beliefs shared any discernible characteristics (e.g. if some themes were only present in Middlesbrough and not South Tyneside, or vice versa). After differentiation, there were few clear differences between the themes present in participant narratives based on any identifiable characteristics. The similarity between participant narratives regardless of background indicates a high degree of consensus among participants and justifies viewing their data as that of a single group.

Prior to the start of their interviews/focus group, participants provided written consent to participate in the study. Several steps were taken to ensure the safety and comfort of participants during their interviews, as DoD can be sensitive topics of discussion. Prior to beginning an interview, participants were advised that if they became uncomfortable during their interview, they could end their participation at any time or ask to skip questions as they saw fit. Most participants completed their interview without becoming upset. In the rare instance where the interviewer observed a participant was distressed by the conversation, they were reminded of their ability to control or end the discussion at any time. Upon conclusion of an interview, participants were provided with a resource sheet with the contact information for local mental health and substance abuse charities that they could contact if they were feeling upset. Participant data is confidential, and anonymised transcripts are not publicly available. Direct quotations used in this publication are not attributed to specific participants in order to mitigate the risk of identification.

## Results

Participants’ narratives contained five distinct themes which highlighted the ways through which austerity had exacerbated DoD in their areas, thereby worsening geographic inequalities. The themes identified in participant narratives were: reduced access to mental health services, loss of specialist substance abuse treatment capacity, loss of youth services, the closure of community institutions, and a growing sense of resentment for the Government.

### Reduced access to mental health services

Waiting lists were identified as a significant barrier to accessing services in both Middlesbrough and South Tyneside. Participants felt that GPs and mental health service providers generally had waiting lists so long as to be unusable to most people. The number of people on waiting lists for healthcare procedures or specialist visits in England has been a matter of public discussion for some time [39]. Although long wait times are characteristic of the healthcare system across England, there are spatial disparities, with more deprived areas such as Middlesbrough and South Tyneside facing longer wait times compared to less deprived regions [40]. Longer waiting times for mental health treatment are associated with worse patient outcomes [41]; and participants in this study provided examples of when these wait times affected their mental health.

“Even your GP. Your GP is hard enough to find now. If you call your GP, they just want to speak to you on the phone. Good luck if you want to actually meet them, you’ll wait years. I had about two mental breakdowns before I had an appointment.” – Middlesbrough Community Member.

In the context of mental health services in England, there is clear evidence that reductions in funding as a result of austerity have reduced access to mental healthcare, resulting in worse mental health outcomes [42, 43]. The wait times that participants identified as a significant barrier to accessing mental healthcare are a direct result of austerity and highlight the importance of considering the role that structural factors, such as availability of services, have in determining mental-health-related outcomes.

Participants believed that when people were unable to access mental health treatment, they turned to mental health support charities. These charities, such as CALM or The Samaritans, were seen by participants to be unhelpful to people in need of support. It was reported that these charities rarely provided meaningful support and had little impact on those who reached out to them. The current evidence on the effectiveness of crisis lines like The Samaritans is limited, showing little indication of improving long-term outcomes and only minimal support for reducing immediate distress in callers [44, 45]. In South Tyneside, community members were concerned about the effectiveness and quality of service delivered by the local crisis service. This service was officially called the Initial Response Service, but community members and many stakeholders referred to it informally as the “crisis team”. These participants believed that the crisis team either did very little for callers, such as telling them to contact their GP, or took extreme action like having people sectioned. Conversely, stakeholders in South Tyneside believed that people misunderstood the purpose of the crisis lines, explaining that while they were intended as a referral service, people in the community expected them to offer immediate mental health support.

“So basically, you speak to a different person each time [you call], so you don’t get to know somebody. I rang up and they said in the end after about 10 or 15 minutes, they asked if I was suicidal. I told them not yet, they said they had people who are suicidal, so they hung up on me. So, basically, I don’t ring them anymore.” – South Tyneside Community Member.

“There is probably a misconception, generally speaking, from the general public about what crisis services do. I think the word “crisis”, that perception is different to everybody. It is very difficult that what some would class as a mental health crisis is very different to what other people would.” There is lots of other things what could be helpful, what could be done in the community. People don’t need to be in hospital, they need a referral.” – South Tyneside Stakeholder.

The disconnect between the services offered by local and national crisis lines (a rapid response to people at immediate risk of taking their own life), and what community members in this study expected these services to provide (ongoing mental health support and care), is emblematic of a larger issue with mental healthcare access caused by austerity; traditional mental health services are inaccessible because of long waiting times caused by inadequate funding. Since these services are largely inaccessible, people turn to crisis lines as they are the only mental health service that they have access to but are unsatisfied because these lines are not equipped to provide ongoing support or psychiatric services. Community members’ negative perceptions of crisis lines and the paucity of evidence supporting their effectiveness suggests that policymakers should pursue other means of improving quick access to mental healthcare, such as reinvestment in community mental health services which provide both crisis response and ongoing mental health support services.

### Loss of substance abuse specialist capacity

Stakeholders believed that the number of drug and alcohol-related services had been reduced in the years following austerity, leaving a service gap that made it difficult for people in need to access support with recovery, thereby increasing drug and alcohol-specific deaths. This view was not expressed by community members. Rehabilitation services were seen to have limited capacity, with very few opportunities for people to do in-patient rehab. The declines in substance abuse service capacity that participants observed are a direct result of changes to the health service funding policy and austerity. The Health and Social Care Act of 2012 transferred public health responsibilities, including funding for substance abuse treatment, from the NHS to local authorities [46]. This coincided with the start of austerity which saw significant declines in local authority budgets [47, 48]. Since 2014/15, there have been significant reductions in spending on substance abuse treatment services [46]. Declining spending on these services has coincided with declines in the number of people accessing services annually and increases in the number of drug and alcohol-related deaths [49, 50]. Stakeholders in this study were acutely aware that the declining capacity of substance abuse treatment services and the corresponding rise in drug-related deaths were a product of austerity.

“You’d be hard pushed to argue the correlation between the substance misuse budget more than halving since we moved from, with the 2013 reforms and public health moved from the NHS to local authorities, over the next sort of 5, 6, 7 years became less than half of what it was. You cannot deny that correlation between disinvestment and the loss of specialist capacity and the drug-related deaths getting to the highest levels on record and Teesside now being one of the drug death capitals of Europe. I just do not think that can be denied or overstated really.” – Middlesbrough Stakeholder.

Concerns about the declining availability of drug and alcohol treatment services were most often expressed by stakeholders, with community members rarely sharing such beliefs. That community members did not identify a decline in funding for drug and alcohol services may speak to a lack of awareness about these services among the general public. Since most people do not need to engage with substance abuse services, they are unlikely to have a high degree of familiarity with the treatment capacity of their local substance abuse treatment organisations.

### Loss of youth services

Participants believed that there was a lack of youth clubs and opportunities for recreation for children and young people. According to participants, youth services had been present at one time but had largely been forced to close in recent years. It was reported that there was simply very little for children to do outside of school hours because there were no youth centres or after-school activities. The closure of youth clubs and services is a direct product of austerity. Austerity brought major reductions in funding from the central government for local councils leading to difficult decisions about which discretionary services could be reduced in order to continue to provide mandatory services such as adult and child care services. Discretionary services including youth clubs, libraries, museums, and parks were heavily reduced or cut altogether as a result [51]. In fiscal year 2011–2012, the start of the austerity era, council spending on services for young people fell from £1184 million to £877 million [51]. The reductions in funding were not applied evenly throughout the country, with the most deprived local authorities, like Middlesbrough and South Tyneside, needing to impose greater funding cuts for youth services than their least deprived counterparts [51], a geographical pattern that was mirrored in other austerity-related funding cuts [47]. Participants in this study were aware that the decline in youth services available in their areas was directly attributable to austerity.

“2010 they were taken away from us. We used to have a big budget for youth provision. So, we would have a lot of, basically local authorities basically funded youth provisions. Youth clubs, council workers. The council employed people with things like degrees and that, so they were professionals, you know? They had an awareness of issues and would be good members of staff to have working with young people. But they took away that funding. The Tories took that away.” – Middlesbrough Community Member.

Participants believed that since there were very few organised activities for young people, and few structured places for them to spend their free time, they would spend their time on the streets; participants felt this left children vulnerable to exploitation, harmed their mental health and led to a rise in substance abuse among young people, thereby increasing the burden of substance abuse and suicide-related deaths.

“When I was younger, there used to be like youth clubs and stuff and community centres. A lot of those are shut down now and there’s not as many facilities for that. I think now that there’s not that, there’s not those facilities, people are just going to go drinking in the field instead, they take drugs and it’s just for something to do really, ‘cause they’ve been told there is no place for them anymore.” – South Tyneside Community Member.

Research in England has found that participation in youth groups provides members with a sense of belonging and social support and improves subjective well-being [52, 53], factors known to reduce the risk of substance abuse and criminal activity among young people [54]. Austerity-related funding cuts to youth services also impacted youth drug and alcohol services, reducing access to education, treatment, and counselling for young people who use substances [55]. By reducing access to youth clubs and youth substance abuse services, austerity both increased the risk of substance abuse among young people and reduced the support available to help treat young people with substance abuse disorders.

“We’ve lost from 2010 to now we’ve lost ground on so many issues because of cuts to local authority budgets and services. We’ve lost the capacity to do some of the things that we have done in the past that we can no longer do. Some of the things we would’ve done, things like our leisure services, our kind of change for life type programmes, youth services, a lot of those things we just can’t fund to the same level. I think you can’t cut those sums of money out of a council budget and expect to deliver the same level of service to people.” – South Tyneside Stakeholder.

### Closure of community institutions

According to participants, services across the board had seen a reduction in funding, which left services in all sectors struggling to meet demand or needing to reduce their service offer. Participants attributed the reduction in funding for services to austerity, reporting that before the austerity measures the service capacity of their areas had been much greater. Services that participants believed had been cut as a result of austerity included educational opportunities for adults, and council services such as refuse collection, street cleaning, and library and community centre opening hours.

“I can tell you now, before 2010, things weren’t like this. There were more youth provisions. There was just generally more support for people. They pulled that funding away and we’re not recovering. It’s getting worse. You see more people on the streets committing antisocial behaviour. We don’t have police officers anymore, you know? They don’t even sweep the streets ‘round here anymore.” – Middlesbrough Stakeholder.

Participants were correct to attribute the decline in council services to austerity-related funding cuts, which have pushed some local authorities to a financial breaking point. Between 2010 and 2020, councils lost more than 50% of their government grants in real terms [56]. Since 2021, six councils have issued a Sect. 114 notice, effectively declaring bankruptcy, and another 14, including Middlesbrough, have indicated that they are at risk of effective bankruptcy in the next year [57]. Participants reported that the lack of council funding for community services had made their areas worse places to live, negatively affected residents’ well-being, and increased social isolation.

“It’s all money driven, and you can only do what you can do, you know what I mean? It’s quite sad. Everything is shut for extra days. You’ve got the town hall. Amazingly that’s only open three days a week. Central Library is shut today, the Grove Hill Library is shut. I was quite stunned at that. The man who founded that library back in the 1800s or whatever, he’d be turning in his grave at this big place of learning shutting half the week. It’s this whole financial thing, but that causes people to become more isolated. I haven’t got that to go to anymore, so people stay in their house.” – Middlesbrough Community Member.

Social isolation was seen as a significant and growing problem in participants’ towns. Participants emphasized the detrimental impact of social isolation on mental health, noting that individuals who lacked social connection often experienced feelings of loneliness and isolation. According to participants, when one was socially isolated or lonely, one’s mental health declined because one had time to ruminate on negative thoughts and feelings, which caused people to turn to drugs and/or alcohol to cope, or suicide to escape. Indeed, loneliness and social isolation are both associated with a wide range of adverse health outcomes and premature mortality [58]. Loneliness has been shown to be associated with increased risk of common mental health disorders like anxiety and depression [59], and increased risk of suicidality independent of the presence of a mental health disorder [60]. Moreover, it increases the risk of harmful and/or dependent drinking and drug use [61, 62]. The importance of opportunities for socialisation and the impact that loneliness has on mental health was discussed during the focus group with community members in South Tyneside. A section of this conversation in which multiple participants discussed this subject is presented below.

**Participant**

It’s socialisation, or not having it. It’s a big thing.

**Participant**

That’s the most important thing.

**Participant**

Every day, no matter what, I just have a walk out to the shops just to get out and see people. If I don’t, I’m just going to sit and watch the same crap on the telly.

**Participant**

True, just to get out.

**Participant**

it’s just a spiral, isn’t it?.

**Participant**

You’ll sit in the house and overthink things. It gets you down.”

**Participant**

Depression, aye. That’s what the guy says to me, that you’ve been on your own too long. You do get depressed on your own.”

Many different kinds of interventions have been developed to combat loneliness and social isolation [63]. Support groups and informal opportunities for group socialisation have proved to be of value in reducing loneliness among adults [59, 63–65]. Unfortunately, as participants explained, many such opportunities have been defunded and closed as a result of austerity measures. This finding indicates the importance of taking a holistic view of loneliness, social isolation, and other risk factors of DoD. It is not sufficient to conclude that people in South Tyneside and Middlesbrough are lonely and that is why some people experience DoD; instead, one must consider the broader structural forces, such as austerity, that give rise to social isolation and loneliness in the first place.

### Resentment for the government

Participants reported that policy decisions like austerity indicated that the central government was out of touch with the needs of towns like Middlesbrough and South Tyneside. Some participants believed the lack of understanding about their towns was a reflection of the North-South divide; this refers to the longstanding geographic health and wealth inequalities between the North and South of England [66, 67]. The root causes of the North-South divide are generally acknowledged to be political and economic [68]. London is both the political and economic centre of England and this has historically influenced economic development policy [68]. Despite efforts by the central government between 2000 and 2010 to reduce regional social and health inequalities [69], the economic and health gaps between the North and South have grown in England since the implementation of austerity [70] and were further exacerbated by the COVID-19 pandemic [71]. Participants believed that austerity and the failure to address inequalities more broadly was because the government did not understand or care that communities in the North had different needs from those in the South, so they passed policies such as austerity that had a disproportionately negative impact on the North.

“Well, just look at the town. It’s not what it used to be, and what it used to be wasn’t exactly great. It’s the same all over the North. I’m blaming these band of Tories but its every politician who ever had power. They look after themselves. They look after their areas. Anything else can just go. There is nothing new in this town as far as I can think of. No schemes to make the town better. They’re just happy to let us all glide along until entropy up here.” – South Tyneside Community Member.

Other participants attributed the out-of-touch nature of the Government to place and class-based stigma. These participants believed that the Government represented the interests of the few and did not care about the needs of impoverished people and places around the country, so they implemented policies like austerity that had a severe impact on already deprived communities. Participants indicated that growing up in a stigmatised community, like Middlesbrough and South Tyneside, instilled a sense of shame and resentment in residents that affected them for the rest of their lives. According to participants, policies such as austerity showed that the Government and broader society did not value people in places like Middlesbrough and South Tyneside. In turn, participants believed that people internalised these beliefs about their community and people living in poverty and believed them to be true about themselves. Participants believed that this negatively affected one’s outlook and general well-being; this belief is consistent with ethnographic research on the effects of territorial stigma elsewhere in the world [72].

Participants believed that feelings of alienation from broader society created resentment for people in other parts of the country. Participants reported that people, particularly those in the South, had no understanding of what life in these areas was really like. The sense of shame described by some participants was visible in other participants’ accounts of their experience living in the community. Participants expressed embarrassment, shame, and anger about the fact that they live in towns that are so looked down upon.

“I’m sorry. I am, I’m sorry I’m from Middlesbrough. I’m ashamed to say I’m from Middlesbrough. That’s why I went to [*a different country*]. If it wasn’t for [*personal circumstances]* I would never have come back here.” – Middlesbrough Community Member.

While participants in this study did not say that territorial stigma had direct impacts on rates of DoD in their areas, the similarity of their accounts of what it is like to live in their community to previous research in other stigmatised places suggests that we can draw inferences about the effects that territorial stigma has on the mental health of people in Middlesbrough and South Tyneside. Territorial stigma has been seen to affect a wide range of mental health-related outcomes, from stress levels [73], to anxiety and depression [74]; factors that participants in this study believed increased the likelihood of one engaging in substance abuse and/or self-harm. Participants’ accounts surrounding the contribution of austerity to place-based stigma suggest that austerity’s impacts were not limited to tangible factors such as reduced access to services and declining availability of mental health services, but that they extended to the social environment and peoples’ conception of self-worth.

## Discussion

The findings of this study align with broader literature on the health consequences of austerity policies in high-income countries. Numerous studies from a range of geographic settings have demonstrated how austerity exacerbates socioeconomic inequalities, leading to worse health outcomes, particularly in disadvantaged areas [75–77]. For example, studies from other contexts, such as the USA and Southern Europe, have similarly found that cuts to welfare, housing, and healthcare services during economic crises correlate with increases in mental health disorders, substance abuse, and mortality rates [78, 79]. The available literature consistently demonstrates the role of austerity measures in shaping health and health inequalities by reducing the safety nets that protect disadvantaged communities. Moreover, participants’ thoughts on austerity’s effect on public services in Middlesbrough and South Tyneside mirror findings from other studies that show how disinvestment in mental health and substance abuse services worsens morbidity and mortality from these causes (Drummond, 2017; Cummins, 2018). The case of DoD in Middlesbrough and South Tyneside reflects the wider understanding in public health literature that austerity not only deepens geographic and class-based health inequalities but also undermines population well-being by eroding the services and supports that are vital to mitigating health risks.

This study makes a novel contribution to the literature by highlighting how austerity has deepened inequalities in DoD in England, a relationship that has been underexplored in previous literature. While much research has focused on the broad health consequences of austerity, this study provides a more focused examination of how austerity policies directly affect DoD in particularly deprived regions like North East England. By drawing on qualitative data from people living and working in Middlesbrough and South Tyneside, this research underscores how reductions in mental health services, substance abuse treatment, community institutions and services, and youth support have compounded geographic health inequalities, leaving already vulnerable populations at greater risk of DoD. This localised focus adds further nuance to the understanding of austerity’s health impacts, showing how structural policy decisions manifest in specific types of mortality within marginalized areas.

The findings of this study provide a clear conclusion that the Government should reinstate the local authority grant at the pre-austerity rate (or indeed, a higher rate due to cost of living increases and inflation) to allow local authorities to reinvest in the services and institutions that were defunded during the austerity era; particularly those related to mental health, substance abuse treatment, basic community services and enrichment, and youth services, which can play a critical downstream role in preventing DoD. By reinvesting in these essential services, the Government can begin to reverse the damage caused by austerity and close inequalities in DoD. Additionally, this study highlights the importance of avoiding further cuts to council and service budgets, as additional disinvestment would likely exacerbate the already critical situation in deprived areas. The evidence presented here supports the broader call for policymakers to prioritise reinvestment in public health and community infrastructure, particularly in communities that have borne the brunt of austerity, to prevent further avoidable suffering and death in these areas.

It is important to note that this paper explores only one factor underpinning DoD in the North East. While participants’ narratives provide a clear picture of the ways through which austerity contributed to the above average rates of DoD present in their towns today, it is not the entire story. DoD are closely tied to factors such as poverty, unstable employment opportunities, and the neoliberal worldview that has shaped policy decisions in Britain and other western countries over the last 40 years [3, 22, 80, 81]. Indeed, the North East’s history of deindustrialisation and the resulting widespread economic precarity and deprivation that persists today creates a unique risk profile that likely explains many of the health inequalities, including those in DoD, that affect the region today [22, 82, 83]. Additionally, the deprivation present in the North East before austerity may help to explain why the impacts of austerity, which were felt to varying extents throughout the country, were disproportionately harmful in the North East. The concept of deprivation amplification asserts that negative health effects of individual deprivation are amplified for those living in more deprived areas [66, 84]. Deprivation amplification likely worsened the effects of austerity on impoverished communities like Middlesbrough and South Tyneside by intensifying existing resource shortages and deepening cycles of poverty. Reinvestment in local services, while a critical first step in preventing DoD, will do little to address the underlying social determinants that create the lived environment that causes suffering and puts people at risk of DoD in the first place. Interventions to address regional inequalities in DoD, and the North-South divide in health more generally, will require a long-term commitment to improving the broader social determinants of health, such as education, economic opportunity, working conditions, and housing.

Research on DoD in the UK has largely used quantitative methods to analyse trends in mortality rates and to examine geographic distributions in morbidity and mortality [9, 13, 15, 85]. While the existing research has provided valuable insight into how DoD are distributed throughout the country, notably absent from the literature are the voices of people living and working in affected areas. It is worth noting that this is a deficiency of the literature surrounding DoD generally, as research on these topics in the US has also been dominated by studies using quantitative methods [86]. This study provides novel findings surrounding how the relationship between DoD and austerity is understood and explained by people living and working in communities experiencing above-average rates of deaths from these causes.

### Strengths and limitations

The findings of this study are grounded in participants’ lived experiences of residing and working in the places in England most affected by DoD. By drawing on lived experiences, this research offers in-depth findings and authentic perspectives that have allowed for a holistic understanding of the ways through which austerity has perpetuated inequalities in DoD in these areas. Additionally, data were collected from a sample that included stakeholders from a broad range of related fields and community members of various ages and backgrounds. This diverse sample enhances the credibility of the findings, ensuring the conclusions are well-rounded and representative of the broad range of perspectives and experiences within these communities. In interpreting the findings of this study, it is important to consider the sample from which they were derived. While efforts were made to engage with a wide range of stakeholders and community members of a range of backgrounds, the recruitment methods used may have failed to reach some communities of people within the case study sites. For example, stakeholders were recruited largely through formal and informal professional networks, which may have inadvertently restricted sampling to only individuals who are well-connected to their peers. There may be groups of stakeholders outside of these peer networks who were not reached by recruitment efforts. Additionally, while the community groups I visited and services I recruited through in Middlesbrough and South Tyneside were open to all residents of their respective areas, these groups may feel unwelcoming to or not be known by people of specific backgrounds or experiences (e.g. refugees and asylum seekers, people who live with a disability, and gender diverse populations) who may hold different views. Additionally, no specific efforts were made to include participants who were closely impacted by DoD (such as by the death of a close friend or family member). Participants with these experiences may view the determinants of these deaths differently than the general population of these towns do.

## Conclusions

This study has demonstrated that stakeholders and community members believe austerity measures have exacerbated inequalities in DoD in vulnerable communities in North East England. Participants identified several mechanisms by which this process occurred; reductions in public funding, especially for mental health services, substance abuse treatment, community institutions, and youth programs, have deepened the vulnerability of these communities, making them more susceptible to the social and economic factors that drive DoD. The widening gap in access to these essential services has further marginalised already struggling populations, reflecting the broader trends of geographic and wealth-based health inequalities in England.

While reinvestment in these services is a necessary first step, it will not be sufficient to completely resolve the inequalities in DoD. Deaths from drug misuse, alcohol, and suicide are symptoms of deeper, systemic inequalities in health and wealth [22]. Therefore, any meaningful intervention must address the broader social determinants of health that give rise to the lived environment in which people experience mental ill-health and engage in harmful substance use. Nonetheless, prioritising the restoration and enhancement of services lost to austerity is critical. Such reinvestment not only helps alleviate some of the most immediate need but also forms a foundation for addressing the wider structural inequalities that perpetuate DoD.

## Data Availability

In order to ensure participant privacy, the data used to generate findings are not publicly available.

## Abbreviations

DoD: Deaths of Despair
